# Determine what to measure and how to measure in clinical trials for the treatment of pressure injury: A protocol for the development of a core outcome set

**DOI:** 10.1097/MD.0000000000019311

**Published:** 2020-02-28

**Authors:** Jiyuan Shi, Ya Gao, Liangliang Si, Xinping Ma, Ming Liu, Xiang Liao, Junmei Zhang

**Affiliations:** a Evidence-based Nursing Center, School of Nursing; b Evidence-Based Medicine Center, School of Basic Medical Sciences, Lanzhou University; c Nursing Department, Henan Provincial People‘s Hospital, Zhengzhou University People's Hospital, Henan University People's Hospital, Zhengzhou, China.

**Keywords:** core outcome set, outcome measurement instruments, pressure injury

## Abstract

**Background:**

A core outcome set (COS) is an agreed minimum set of outcomes that should be reported in all clinical trials in specific areas of health care. A considerable amount of trials did not report essential outcomes or outcomes measurement methods, which makes it challenging to evaluate the efficacy and safety of treatment strategies for pressure injury (PI) and produced significant heterogeneity of reported outcomes. It is necessary to develop a COS, which can be used for clinical trials in PI treatment.

**Methods/Design:**

The development of this COS will be guided by an advisory group composed of clinicians, senior nurses, patients, and methodologists. We will search six databases and 2 registry platforms to identify currently reported PI treatment outcomes and outcome measurement instruments in randomized controlled trials, meta-analysis, and systematic reviews. We will also conduct a semi-structured interview with clinicians, nurses, and adult PI patients to collect their opinions on important outcomes. Each outcome of the initial list generated from systematic review and interviews will be scored and reach a consensus through two rounds of international Delphi survey with all key stakeholders. A face-to-face consensus meeting with key stakeholders will be conducted to finish a final COS and recommend measurement instruments for each outcome.

**Results:**

We will develop a COS that should be reported in future clinical trials to evaluate the effectiveness of PI treatment.

**Discussion:**

The COS will follow current guidance to develop a high-quality COS in the field of PI treatment to reduce heterogeneity in trial reporting, facilitate valid comparisons of new therapies, and improve the quality of clinical trials.

## 1 Introduction

Pressure injury (PI) is localized damage to the skin and underlying soft tissue, usually over a bony prominence or related to a medical or other device.^[1]^ PI prevalence (categories 1–4) ranges from 8.8% to 29.9% in nursing homes ^[2–5]^ and between 7.3% and 23.0% in hospitals throughout Europe and North-America.^[6–9]^ The daily cost of PI treatment per patient ranged from 1.71 € to 470.49 € across different settings.^[10]^ PI has been a common problem faced by global health care institutions, which seriously threat patients’ life and health; it brings heavy economic pressure and medical burden to society.

In recent years, researchers have carried out a large number of studies on risk factors, treatment, prevention, and diagnosis of PI to provide support for the prevention and treatment of PI. However, the outcomes for the therapeutic effect on PI were measured in various ways, and there were little standards across studies.^[11]^ Some clinical trials did not report essential outcome indicators, such as time to complete healing, the proportion of PI healed, healing rate and adverse events.^[12–18]^ When a systematic review (SR) was conducted, the review's prespecified outcomes were hard to obtain in the included trials.^[11,19]^ There is also a lack of reports of PI treatment outcome measurement instruments (OMIs), and the same outcomes were measured by different methods in clinical trials ^[17,18,20]^ or measured in differing time of follow-up.^[16,17,21–23]^ Only a handful of studies considered PI patients’ perspective of which outcomes should be reported in clinical trials of PI treatment, and this may undermine the external validity of published research in clinical practice.^[24,25]^

Choosing appropriate outcome indicators can not only improve the reliability and practicability of clinical research, but also promote the transformation of clinical research results into clinical practice.^[26]^ However, there are significant differences in the reporting forms of PI results, which will bring inconvenience to the statistical analysis of the results and hinder the formulation of clear evidence-based treatment recommendations for PI. This problem may be solved by developing a core outcome set (COS), which is an agreed and minimal set of outcomes that should be measured and reported in all clinical trials in specific areas of health or healthcare.^[27]^

The overall aim of our research is to develop a COS that should be reported in future clinical trials to evaluate the effectiveness of PI treatment interventions in adults and select a measurement instrument for each outcome. It is essential to consider all key stakeholders’ perspectives on the importance of outcomes when conducting a core outcome set, and we will develop this COS followed by the recommendation from the COMET handbook (version 1.0) ^[24]^ and the Core Outcome Set-STAndards for Reporting (COS-STAR).^[25]^

## 2 Materials and methods

**Figure 1:**
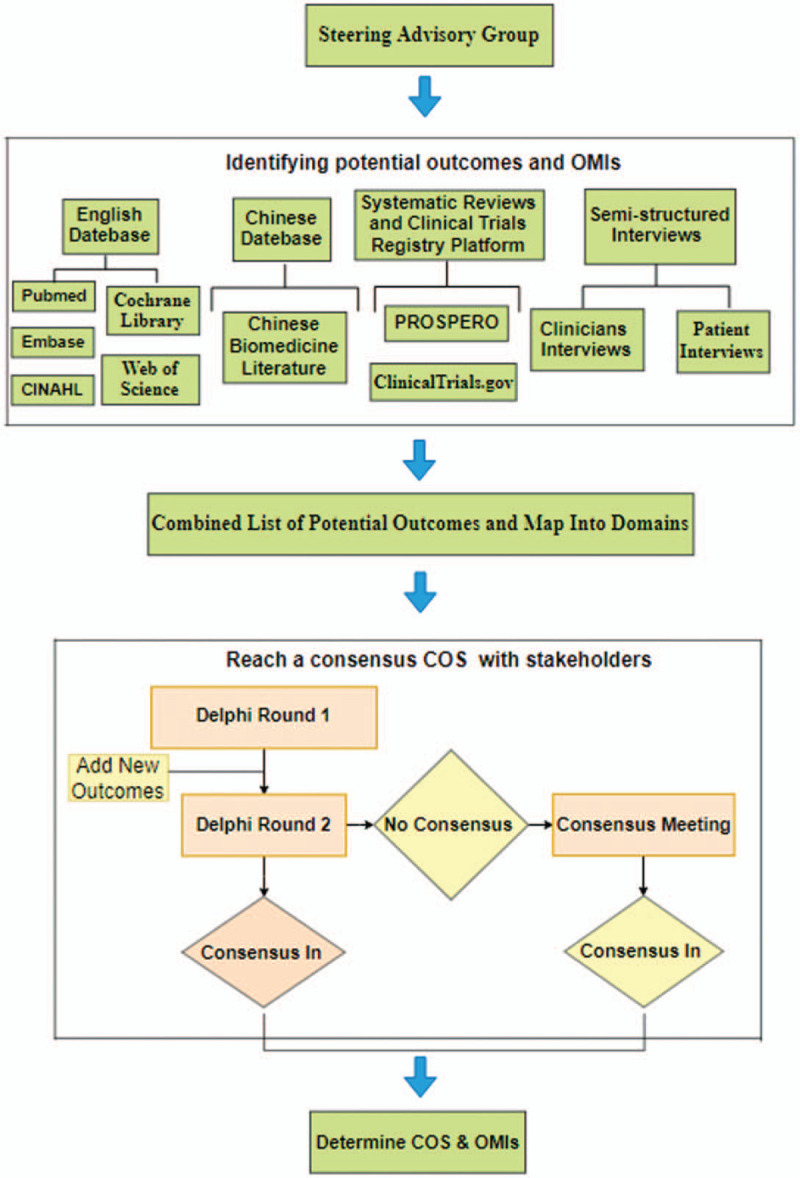
An overview of core outcome set (COS) project.

We will develop the COS of PI treatment based on the general guidelines of the COMET handbook,^[24]^ COS-STAD^[25]^ and consensus-based standards for the selection of health measurement instruments (COSMIN)^[28]^. An overview of our studies’ progress is shown in Figure 1.

### 2.1 Registration

Our research has been registered on the COMET initiative (http://cometinitiative.org/studies/details/1408).

### 2.2 Steering advisory group

A steering advisory group will be created, including clinicians, senior nurses, patients, and methodologists. The group will evaluate the preliminary checklist of the outcome, and add important outcome if they think which have been left off the checklist. The advisory group will take part in the consensus meeting to develop the COS.

### 2.3 Stage 1: Systematic review (previously reported outcomes).

#### 2.3.1 Literature search

We will conduct a comprehensive search of PubMed, Embase, Cochrane library, Web of Science, the Cumulative Index to Nursing and Allied Health Literature, Chinese Biomedicine Literature, International Prospective Register of Systematic Reviews (PROSPERO) and ClinicalTrials.gov to identify relevant clinical trials and SRs. Titles and abstracts of publications are selected independently by two reviewers (JYS and YG). The same two reviewers will retrieve full-text of potentially eligible studies and determine study inclusion or exclusion independently. Any disagreement will be resolved by a discussion with a third reviewer (ML).

#### 2.3.2 Eligibility and inclusion

Studies will be included if:

- all randomized controlled trials (RCT), meta-analysis, and SRs that reported any type of PI treatment in adult (aged≥18 years) will be included
- all reported outcomes will be included.

Studies will be excluded if:

- review, abstracts and letters will be excluded
- the main objectives of the study were to prevent PI will be excluded
- for feasibility, we will exclude articles published in languages other than Chinese and English.

#### 2.3.3 Data extraction and analysis

The data will be extracted from each study by 2 reviewers (XM and YG) independently. The data items include first author name, journal, number of participants, study type, time-point, intervention details, effectiveness, and safety outcome(s), follow-up, and outcome measurement instruments. Any disagreement will be discussed with a third reviewer (ML). The data will then be grouped into appropriate outcome domains defined by the COMET handbook,^[24]^ and the advisory group will confirm the classification and outcome list. The frequency of each outcome and the method of outcome measurement will be recorded.

### 2.4 Stage 2: Semi-structured interviews

#### 2.4.1 The inclusion/exclusion criteria of stakeholders

According to recommendations of COS-STAD and COMET handbook (version 1.0),^[24,25]^ it is necessary to obtain the opinion from stakeholders on PI treatment. Semi-structured interviews will be conducted to acquire stakeholders’ perspectives about the outcomes of treating PI that should be measured in a clinical trial.^[29]^ This project will facilitate us to understand which outcomes are patients, clinicians, and nurses focus on, and further refine our list of results. The inclusion/exclusion criteria of patients, clinicians, and nurses are shown in Table 1.

**Table 1:**
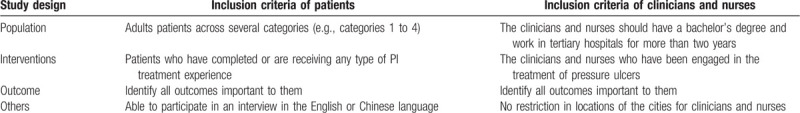
The inclusion/exclusion criteria of patients, clinicians, and nurses.

#### 2.4.2 Sampling

There are no robust standards for the sample size of semi-structured interviews. We will divide participates into the patient group and the clinical group. More than 20 patients will be recruited, and we will recruit patients in a diversity of age, sex, treatment types, and PI categories following the purpose of obtaining the overall outcomes. We will also invite 20 clinicians and 20 nurses who meet our criteria list in Table 1.

#### 2.4.3 Data collection and analysis

The analysis of the data will be conducted simultaneously with the data collection. Investigators will explain the purpose of this study to participates, they can withdraw at any time, and participants who have completed the survey will be invited to continue to participate in the Delphi survey and consensus meeting as a representative.^[30]^ A face-to-face conversation will be conducted after all informed consents are signed, questions in English and Chinese will be provided according to the choice of participants, and each investigator will be trained before. All participates will review the outcome list generating from the systematic review, and we will use open questions as a topic guide. All the interviews will be audio-recorded, the semi-structured interview will be conducted until the thematic saturation, and no new outcome is obtained. We will use qualitative analysis software (NVivo 11, QSR International Pty Ltd., Burlington, MA) to import the recordings and analyze through thematic analysis by the framework method. Researchers and the Steering Advisory Committee will identify whether these outcomes are new and judge whether they should be added to the list of candidate outcomes.

### 2.5 Stage 3: Delphi survey

Our original list will go through two rounds of questionnaires by using the Delphi Method for optimization,^[31]^ and each series of questionnaires will last 2 to 3 weeks. If the response rate is too low, the time of the following questionnaires recycling can be prolonged appropriately.

#### 2.5.1 Stakeholders involvement

The participation of stakeholders on a broad geographic scale, and multiple groups will be invited, including clinicians, senior nurses, patients, researchers. However, there are no robust standards for the sample size of Delphi survey, and we will use snowball sampling to expand the scale of stakeholders, the experts will invite colleagues who they think meet the criteria for inclusion in the study. In the first round, each stakeholder group will be invited at least 20 participants.

#### 2.5.2 Round one

We will invite participants through email with links to the original questionnaire. Before carrying out the survey, all Delphi survey texts will be approved by the advisory group to confirm the readability of the language. Each participant will use the 9-point Likert-type scale to rate each outcome from 1 (unimportant) to 9 (critically important outcome), depending on the importance of each item. In the first round of Delphi questionnaires,^[32–34]^ participants can put forward new items that may be included in the second round of questionnaires.^[35,36]^

#### 2.5.3 Round two

Participants who have completed round 1 of the Delphi survey will be invited to round 2. The number of participants who have scored each item and the score they rate the outcome in round 1 will show to participants. All stakeholder groups will be asked to re-score each outcome. As shown in Table 2, each outcome will be defined as 3 categories.^[35,36]^

**Table 2:**
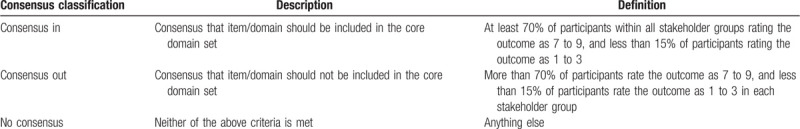
Definition of consensus.

### 2.6 Stage 4: Consensus meeting

After completing the Delphi survey, each outcome scored from the Delphi survey will be present at face to face meetings to reach a final consensus about a COS that should be reported in all clinical trials of PI treatment with COMET guidance.^[24]^ Participants who have participated in the two rounds of Delphi survey will be requested to attend face to face meetings. We aim to gather approximately 15 to 20 participants with equal representation from each stakeholder group. Journal editors and policymakers will also be invited. After discussion, participants will be required to score each of “no consensus” outcome by the anonymous method according to the same scoring system as the Delphi process, and for which consensus is achieved in at least one but not all stakeholder groups, further discussion will take place. Outcomes achieving “consensus in” will be included in the final COS. After finishing the final COS, participants will use the same scoring system to discuss and recommend the time point of measurement and OMIs for every included outcome.^[24,27]^ The meeting will also discuss and reach an agreement on dissemination strategies.

## 3 Dissemination

The finished COS will follow recommendation of recent studies on dissemination strategies,^[35–37]^ All outcomes of each step, including excluded outcomes will be published in a peer-reviewed journal, each of participates of the COS will be asked to implement the COS in their future clinical trials of PI treatment and recommend this COS to their colleagues and other potential researchers. We plan to present our results at international conferences and post our final COS information on the COMET website. We will also send our article to professional associations and related groups by e-mail if our manuscript is accepted.

## 4 Discussion

PI has been a common problem faced by global health care institutions,^[19]^ which seriously threatens the life and health of patients. It is uncertain which treatment strategy is more clinically effective and more acceptable to PI patients, or more cost-effective compared to others. There are still a significant number of clinical trials in varies of therapeutic methods are being performed, but there are almost no standards in reporting outcomes for the therapeutic effect on PI across clinical trials.

The lack of standards reporting on outcomes of the clinical trial may cause heterogeneity and hinder evidence synthesis in systematic review/meta-analysis.^[24,27,38]^ After years of development, there are more researchers focus on the importance of developing a COS, but there are still a great number of clinical trials in PI treatment that have been conducted without a COS currently exists or is in development.^[39]^ A high-quality COS can help combine and compare the result of trials to reduce waste in research, and it is necessary to improve the quality of trials result by developing a COS of PI treatment. It is worth noting that COS is not to limit outcomes reporting; it just represents the minimum outcomes that should be measured and reported, researchers can still explore reporting other appropriate outcomes.^[25]^

The main objective of this project is to follow current guidance to develop a high-quality COS in the field of PI treatment, including all efficacy and safety outcomes and measurements as determined. Furthermore, if researchers select a COS of PI treatment developed by high standards, it can reduce the reporting bias of clinical trials and promote evidence-based recommendations.

## Acknowledgments

We express our thanks to Jinhui Tian for the the assistance with the design of the protocol.

## Author contributions

JS, JZ conceived the idea for this study; JS, LS and XM designed the core outcome set; ML provided statistical advice and input; JS and YG drafted the protocol. JZ and XL reviewed the protocol and provided critical feedback. All authors approved the article in its final form.
